# Comparison of three commercial multiplex PCR assays for the diagnosis of intestinal protozoa

**DOI:** 10.1051/parasite/2018049

**Published:** 2018-09-18

**Authors:** Brice Autier, Sorya Belaz, Romy Razakandrainibe, Jean-Pierre Gangneux, Florence Robert-Gangneux

**Affiliations:** 1 Laboratoire de Parasitologie-Mycologie, Centre Hospitalier Universitaire de Rennes 2 rue Henri Le Guilloux 35033 Rennes France; 2 Laboratoire de Parasitologie-Mycologie, CNR Laboratoire expert Cryptosporidiose, Centre Hospitalier Universitaire de Rouen Rouen France

**Keywords:** multiplex PCR, intestinal protozoa, *Giardia intestinalis*, *Entamoeba histolytica*, *Cryptosporidium* sp., *Dientamoeba fragilis*

## Abstract

Although microscopic examination of stool samples remains the reference method for the diagnosis of intestinal protozoal infections, these techniques are time-consuming and require operators who are experienced and well trained. Molecular biology seems to offer performances at least equivalent in terms of sensitivity and specificity for certain parasites. This study aimed to compare three multiplex PCR assays on 93 prospectively collected positive stools (prospective cohort) and a panel of 12 more *Cryptosporidium*-positive samples (*Cryptosporidium* panel). On the prospective cohort, the sensitivity was 89%, 64% and 41% for *Giardia* sp. detection for BD Max^TM^, G-DiaPara^TM^ and RIDA^®^GENE, respectively and 75%, 100% and 100% for *C. parvum/hominis* detection. The sensitivity of the RIDA^®^GENE assay for all *Cryptosporidium* species was 100%, and for *D. fragilis* 71%. All the techniques obtained the same results for *E. histolytica* detection, with one positive sample. All species in the *Cryptosporidium* panel were identified by the RIDA^®^GENE PCR. The BD Max^TM^ and G-DiaPara^TM^ assays detected only *C. parvum/hominis* with the exception of one positive sample for *C. meleagridis*. No assay showed satisfactory results for all parasites simultaneously, and the DNA extraction seems to be the critical step. More studies are needed to standardize this procedure.

## Abstract (fr)

Bien que l’examen microscopique des selles reste la méthode de référence pour le diagnostic des protozooses intestinales, ces techniques sont chronophages et demandent une grande expérience et des opérateurs entrainés. La biologie moléculaire semble offrir des performances au moins équivalentes en termes de sensibilité comme de spécificité pour certains parasites. Cette étude visait à comparer trois techniques de PCR multiplex sur une cohorte de 93 selles positives collectées prospectivement et un panel de 12 échantillons positifs à *Cryptosporidium*. Respectivement pour BD Max^TM^, G-DiaPara^TM^ et RIDA^®^GENE la sensibilité était de 89 %, 64 % et 41 % pour la détection de *Giardia* sp. et 75 %, 100 % et 100 % pour la détection de *C. parvum/hominis*. La sensibilité de la technique RIDA^®^GENE pour l’ensemble des espèces de *Cryptosporidium* était de 100 % et de 71 % pour *D. fragilis*. Toutes les techniques ont obtenu les mêmes résultats pour la détection d’*E. histolytica* (1 échantillon positif). Toutes les espèces de *Cryptosporidium* ont été détectées par la PCR RIDA^®^GENE. Les techniques BD Max^TM^ et G-DiaPara^TM^ ont détecté seulement *C. parvum/hominis* en dehors d’un échantillon positif à *C. meleagridis*. Aucun essai n’a montré de résultats satisfaisants pour l’ensemble des parasites simultanément et l’extraction d’ADN semble être l’étape critique. Plus d’études sont nécessaires afin de standardiser cette procédure.

## Introduction

Because the world is in the era of globalization, laboratories are confronted with a growing number of parasitic diseases affecting people living in endemic areas, migrants, travellers and international workers [19]. Intestinal protozoan parasites are responsible for a broad spectrum of clinical manifestations, ranging from mild gastrointestinal symptoms to life-threatening watery or haemorrhagic diarrhoea, and can even lead to complications with extra-intestinal localizations. Among them, giardiasis and dientamoebiasis are a major cause of disease in terms of frequency [1, 22], and cryptosporidiosis and amoebiasis are, respectively, the third and fourth parasitic causes of death worldwide [8]. However, these infections are often neglected and underreported.

Diagnosis of protozoan intestinal infections mainly relies on microscopic examination after stool concentration, which remains the reference method, as it enables detection of all parasites. However, it is time-consuming and requires special skills and well-trained operators, since protozoan parasites are somewhat difficult to identify, particularly when they are present in low numbers. Therefore, new diagnostic methods are needed. While the development of molecular techniques marked a turning point in the diagnosis of bacterial and viral diseases, these techniques are more difficult to apply to the diagnosis of intestinal protozoosis, due to the thick wall of parasite (oo)cysts, making DNA extraction difficult, and due to the high density of PCR inhibitors in stool samples. Many procedures have been developed, often based on pre-treatment of stool samples by mechanical, chemical or thermic lysis, and followed by various extraction systems, like silica-column-based or magnetic extraction, with variable performances. Today, molecular biology techniques could help to save time, provided that they are able to detect in a same run most protozoan parasites infecting humans, and that they can be automated.

In this study, we evaluated three commercial multiplex PCR assays, BD Max^TM^ Enteric Parasite Panel (Becton Dickinson, Pont-de-Claix, France), G-DiaPara^TM^ (Diagenode Diagnostics, Liege, Belgium), and RIDA^®^GENE Parasitic Stool Panel I (R-BioPharm, Darmstadt, Germany), and compared them to microscopy, using the same panel of stool samples found positive for protozoa by microscopic examination. The BD Max^TM^ assay is fully automated, and the two other methods were evaluated after automated extraction using MagNA Pure 96 (Roche Diagnostics, Meylan, France), with the aim of adapting amplification steps on a flow system (Roche).

## Materials and methods

### Stool samples

**Figure 1.**
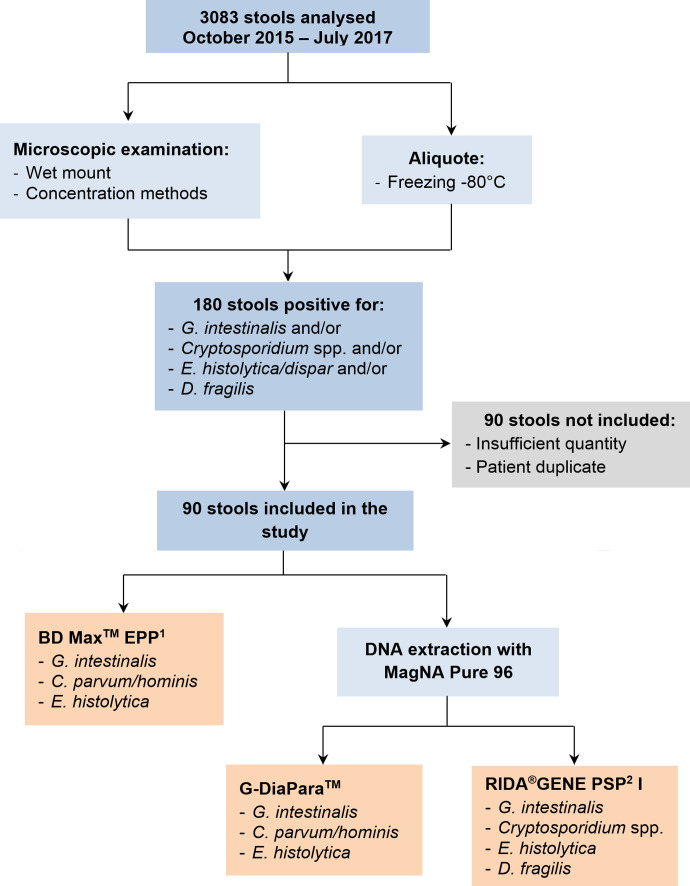
Study flow chart. ^1^EPP: Enteric Parasite Panel; ^2^PSP: Parasitic Stool Panel.

This study included 90 stool samples analysed prospectively at the Consultation Unit of the Laboratory of Parasitology of the University Hospital of Rennes (France) over an 18-month period (October 2015–July 2017) and tested positive for *Giardia intestinalis*, *Cryptosporidium* sp., *Entamoeba histolytica/dispar* and/or *Dientamoeba fragilis* by microscopic examination (Fig. 1), so-called “prospective cohort”. Three additional samples positive for commensal protozoa and/or helminths were also included in order to assess the specificity of the assays. These 93 samples were analysed by experienced operators, as quickly as permitted by the opening hours of the laboratory and the timeframe for transport of the stool. The procedure consisted in a microscopic examination of a fresh stool wet mount and in-house concentration methods (Bailenger’s, Thebault’s and merthiolate-iodine-formalin biphasic methods [5]). *Cryptosporidium* detection relied on Henriksen’s modified Ziehl-Neelsen staining on concentration pellets. An aliquot of stool sample was also frozen at −80 °C until DNA extraction. When *Cryptosporidium* sp. oocysts were detected, a stool sample was sent to the French National Reference Centre for Cryptosporidiosis (NRCC) (University Hospital of Rouen, France) for species identification by sequencing analysis. For all samples, parasites were semi-quantified as “rare”, “few”, “quite numerous”, “numerous” and “many”. Twelve samples provided by the NRCC were also included to extend our cohort with stool samples positive for various species of *Cryptosporidium* (“*Cryptosporidium* panel”). These specimens were stored frozen with potassium dichromate 2.5%. The final stool panel consisted of 44 *G. intestinalis*, 23 *Cryptosporidium* spp. (13 *C. parvum*, 5 *C. felis*, 3 *C. hominis*, 1 *C. canis,* and 1 *C. meleagridis*), 13 *E. histolytica/dispar*, and 28 *D. fragilis*. Six samples were poly-parasitized: 2 with *D. fragilis* and *G. intestinalis*, 2 with *E. histolytica/dispar* and *G. intestinalis*, and 2 with *Cryptosporidium* sp. and *G. intestinalis*.

### DNA extraction using MagNA Pure 96

Stool DNA was extracted with BD Max^TM^ (Becton Dickinson, Franklin Lakes, NJ, USA) and MagNA Pure 96 (Roche Diagnostics, Meylan, France). For each sample, a small amount (approximately pea-sized) of thawed stool was suspended in 500 μL of PBS and vortexed, except for liquid stools, which were used plain. In case of insufficient quantity of stool, the volume of PBS was reduced proportionally. Suspensions were centrifuged for 5 s at 500 *g*, and 90 μL of supernatant were added to 90 μL of MagNA Pure 96 Bacteria Lysis Buffer (Roche Diagnostics) and 20 μL of proteinase K (Roche Diagnostics). After homogenization, the mix was incubated for 10 min at 65 °C, followed by 10 min at 95 °C. Then, two cycles of freezing and thawing were performed (at least 10 min at −80 °C followed by 10 min at 95 °C). Each incubation step was followed by vortexing to homogenize the suspension. After a short centrifugation (few seconds at max speed), 20 μL and 10 μL of the RIDA^®^GENE Parasitic Stool Panel I (PSP I) (R-BioPharm) and DiaControlDNA^TM^ Cy5 (Diagenode Diagnostics) internal controls, respectively, were added. Mixes were then extracted with a MagNA Pure 96 system (Roche Diagnostics Ltd) using MagNA Pure 96 DNA and Viral NA Small Volume (Roche Diagnostics Ltd), and eluted in a 100 μL volume. Other pre-treatment procedures were tested on 17 samples: one with a bead-beating step before thermal and chemical lysis, and another with only chemical lysis. Bead-beating was done with MagNA Lyser Green Beads (Roche Diagnostics) using the MagNA Lyser device (Roche Diagnostics Ltd), for 35 sec at maximum speed and repeated twice, followed by centrifugation for 1 min at 8000 *g*, before lysing the supernatant as described above. DNA extracts were stored at −80 °C until amplification with the RIDA^®^GENE PSP I and the G-DiaPara^TM^ assays.

### Amplification

DNA extracts were amplified by the RIDA^®^GENE PSP I (R-BioPharm) and G-DiaPara^TM^ (Diagenode Diagnostics) assays using a LightCycler 480 II device (Roche Diagnostics), following the manufacturer’s recommendations. As *D. fragilis* can be detected only by RIDA^®^GENE PSP I, a total of 65 and 93 samples were analysed with the G-DiaPara^TM^ and the RIDA^®^GENE PSP I assays, respectively. The pathogens possibly detected by each assay are shown in Figure 1. Briefly, for the RIDA^®^GENE PSP I assay, 5 μL of DNA were added to 19.9 μL of mix and 0.1 μL of Taq polymerase solution, and amplified for 45 cycles with a 15 s extension step at 60 °C. For the G-DiaPara^TM^ assay, 5 μL of DNA were added to 20 μL of mix composed of 5 μL of Master Mix 5X (Diagenode Diagnostics), 2.5 μL of probes and primers for parasite detection, 2.5 μL of probes and primers for internal control detection, and 10 μL of water suitable for molecular biology. Amplification was carried out for 45 cycles with an extension step at 60 °C for 60 s. Positive and negative controls were included in each run on the LC 480 II (provided by PCR kits). False-negative results with the RIDA^®^GENE PSPI and G-DiaPara^TM^ assays were re-analysed using DNA extracts diluted at 1:10. For two stool specimens (1 *C. parvum* and 1 *C. meleagridis*), the remaining quantity was insufficient for extraction on MagNA Pure 96, thus PCR assays were performed using BD Max^TM^ extracts stored at −80 °C.

### Extraction and amplification with the BD Max^TM^ system

A small quantity of thawed stool was collected with a 10 μL-calibrated loop and suspended in a tube containing 1.5 mL of lysis buffer, supplied in the BD Max^TM^ EPP kit. Tubes were heated for 52 min in a pre-warmed heater device, before being transferred into a BD Max^TM^ system rack containing unitized dried reagents. Processing by the BD Max^TM^ system includes a DNA extraction step with magnetic beads and multiplex amplification using Taqman^®^ technology. Each rack allows the analysis of up to 24 samples in a same run. Positive controls (positive clinical samples) were included in the first run only. As with the G-DiaPara^TM^ assay, 65 samples were analysed with this technique (exclusion of stools positive for *D. fragilis* only). After analysis, all remaining extracts were stored at −80 °C.

### Statistics

Microscopy was considered as the reference method to calculate the sensitivity and specificity of each PCR assay.

## Results

### Impact of the pre-treatment method on PCR performance

As shown in Table 1, the bead-beating pre-treatment inhibited the detection of *Cryptosporidium* spp. in the selected samples. The detection of *G. intestinalis* was moderately affected, while *E. histolytica* and *D. fragilis* detection was apparently not affected, with the limitation of the small number of samples. Freezing and thawing cycles were maintained because of better amplification of internal controls (C_T_ from 25–29 versus 30–33 without, data not shown), even if the number of positive stools was not impacted.

**Table 1.** Results for tested pre-treatments.

	Results by RIDA^®^GENE assay (*n*/*N*)	
	Without bead-beating	With bead-beating
*G. intestinalis*	5/8	3/8
*Cryptosporidium* sp.	4/5	0/5
*E. histolytica*	1/1	1/1
*D. fragilis*	2/3	2/3

### Evaluation of the 3 PCR assays’ performances

**Figure 2.**
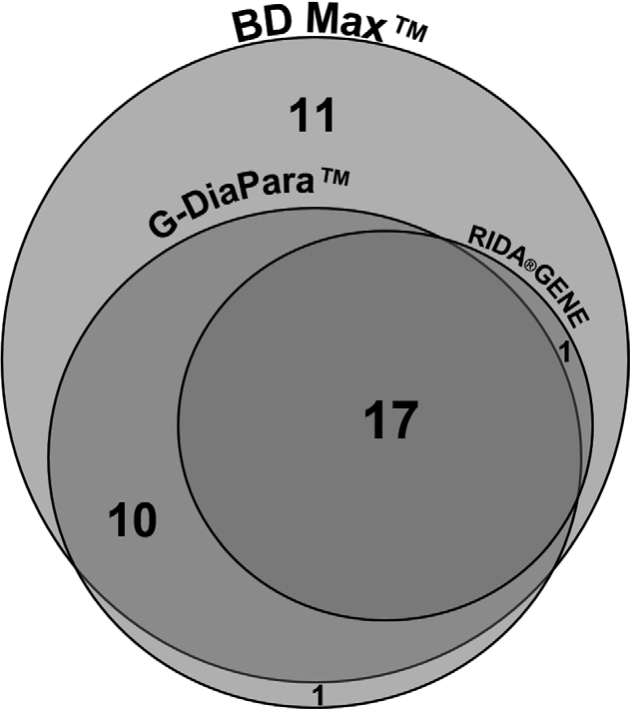
Venn diagram of results for *Giardia* detection. All *Giardia*-positive samples detected by the RIDA^®^GENE assay were detected by the G-DiaPara^TM^ and the BD Max^TM^ assays, and almost all those detected by the G-DiaPara^TM^ assay were detected by the BD Max^TM^ assay (1 exception).

The detection of *Giardia* by the BD Max^TM^, G-DiaPara^TM^ and RIDA^®^GENE assays yielded respectively a sensitivity of 89%, 64% and 41%, respectively (Table 2). False-negative results were obtained with samples showing “rare” or “few” parasites by microscopy, except for 4 which were negative by G-DiaPara^TM^ and/or RIDA^®^GENE PSP I assays while quantified as “quite numerous” or “numerous” by microscopy. Five false-negative samples were obtained with the BD Max^TM^; in none of them the results of the internal control suggested the presence of PCR inhibitors. All three assays detected a positive stool which was negative by microscopy, leading to a specificity of 95%–98%. As illustrated in Figure 2, samples tested positive with the RIDA^®^GENE PSP I assay were also positive with the G-DiaPara^TM^ and BD Max^TM^ assays, and almost all samples positive with the G-DiaPara^TM^ assay were also positive with the BD Max^TM^ assay.

**Table 2.** Performances of the multiplex PCR assays compared to microscopy on 93 positive samples analysed routinely na: not applicable; nd: not determined.

	Sensitivity *n*/*N* (%)			Specificity *n*/*N* (%)		
	BD Max^TM^	G-DiaPara^TM^	RIDA^®^GENE	BD Max^TM^	G-DiaPara^TM^	RIDA^®^GENE
*G. intestinalis*	39/44 (89%)	28/44 (64%)	18/44 (41%)	20/21 (95%)	20/21 (95%)	48/49 (98%)
*Cryptosporidium* sp.	6/11 (55%)	8/11 (73%)	11/11 (100%)	54/54 (100%)	54/54 (100%)	82/82 (100%)
*C. parvum/hominis*	6/8 (75%)	8/8 (100%)	8/8 (100%)	na	na	na
*D. fragilis*	na	na	20/28 (71%)	na	na	63/65 (97%)
*E. histolytica/dispar*	nd^1^	nd^1^	nd^1^	nd^1^	nd^1^	nd^1^

1 As microscopy does not enable species identification, sensitivity and specificity could not be determined for *E. histolytica* detection. However, concordant positive results were obtained with the 3 PCR assays for 1 sample.

The sensitivity for *Cryptosporidium* sp. detection by BD Max^TM^, G-DiaPara^TM^ and RIDA^®^GENE PSP I assays was for the prospective cohort 55%, 73% and 100%, respectively. When considering only samples positive for *C. parvum* and *C. hominis,* as targeted by their respective primers, the first two assays reached sensitivities of 75% and 100%, respectively. In this prospective cohort, the RIDA^®^GENE assay detected all positive stools, the G-DiaPara^TM^ assay detected all *C. parvum/hominis*, and the BD Max^TM^ assay did not detect two stools positive for *C. parvum/hominis* because of PCR inhibitors (Table 2). Specificity was excellent for all techniques (100%). When considering the 12 samples from the NRCC *Cryptosporidium* panel (Table 3), the RIDA^®^GENE assay missed two *Cryptosporidium* sp. (one *C. hominis* sample containing PCR inhibitors and one *C. felis*), the BD Max^TM^ assay missed two *C. hominis* positive stools, and G-DiaPara^TM^ detected all *C. parvum/hominis*. None of the G-DiaPara^TM^ and BD Max^TM^ assays detected the other species except *C. meleagridis*. For three of the four false-negative results by the BD Max^TM^ assay, the internal control highlighted PCR inhibitors. The RIDA^®^GENE assay detected at least one sample for each *Cryptosporidium* species tested, confirming the wide detection of *Cryptosporidium* species.

**Table 3.** Results for the *Cryptosporidium* sp. panel from the French NRCC.

	Sensitivity: *n*/*N*		
	BD Max^TM^	G-DiaPara^TM^	RIDA^®^GENE
*Cryptosporidium* sp. (*N* = 12)	7/12	9/12	10/12
*C. parvum* (*N* = 5)	5/5	5/5	5/5
*C. hominis* (*N* = 3)	1/3	3/3	2/3
*C. felis* (*N* = 2)	0/2	0/2	1/2
*C. canis* (*N* = 1)	0/1	0/1	1/1
*C. meleagridis* (*N* = 1)	1/1	1/1	1/1

Because microscopy cannot be used to identify *E. histolytica/dispar* species, neither sensitivity nor specificity could be calculated. However, results were identical for the three assays, which detected one sample positive for *E. histolytica* among the stools positive for *E. histolytica/dispar*. Another sample was collected from the same patient 4 days before this one (not included): microscopic examination showed only commensal protozoa (*Entamoeba coli* and *Blastocystis hominis*) but all PCR assays were positive for *E. histolytica*.

Among the 28 stools positive for *D. fragilis*, 20 tested positive with the RIDA^®^GENE assay, yielding a sensitivity of 71%. Among the eight false-negative samples observed, all were quantified as “rare” or “few”, except one quantified “quite numerous” by microscopy. No PCR inhibitor was detected by internal controls. Two samples negative by microscopy were positive by the RIDA^®^GENE assay (specificity 97%), one contained *Endolimax nanus*, *Chilomastix mesnili* and *Pentatrichomonas intestinalis*, and the other contained *Blastocystis hominis*, *G. intestinalis* and *C. mesnili*.

PCR inhibitors were detected in 26% of stools by the BD Max^TM^ assay (17/65), impacting only detection of *Cryptosporidium sp*., and 0% (0/65) and 1% (1/93) for the G-DiaPara^TM^ and RIDA^®^GENE assays, respectively. The extract with inhibitors detected by the RIDA^®^GENE assay was associated with a false-negative result for *Giardia* sp. The amplification of diluted extracts did not enable recovery of false-negative results.

## Discussion

The aim of this study was to evaluate and compare three PCR assays for the detection of intestinal protozoa. Performances obtained on our prospective cohort of 93 consecutive frozen stool samples from 93 patients, positive by microscopic examination, were heterogeneous among assays and targets. Overall, we observed high specificity, but variable sensitivities for *Giardia intestinalis* and *Cryptosporidium* sp. detection. BD Max^TM^ was the most sensitive for *G. intestinalis* detection (89%), followed by G-Diapara^TM^ (64%) and RIDA^®^GENE (41%). These two latter sensitivities are poorer than previously observed in other studies. Indeed, Laude et al. observed 92% sensitivity with the G-DiaPara^TM^ assay on a smaller series of 38 *Giardia*-positive stool samples after extraction with the QiaSymphony device (Qiagen, Courtaboeuf, France) [12]. To our knowledge, there are no evaluations of the performances of the RIDA^®^GENE Parasitic Stool Panel I in the literature, but similar panels from the same manufacturer showed sensitivities of 95%–100% in some reports [13, 15, 21]. Nevertheless, in these reports, only few data are available about the microscopic examination performed: it is therefore difficult to interpret the observed performances. It is well known that the extraction process influences the results of amplification. These disappointing results cannot therefore be solely attributed to the PCR assay. As discussed further, even though we tried to optimize DNA extraction from cysts by using various pre-treatment protocols, many factors involved in this step could interfere in our study. However, it can be stated that the G-DiaPara^TM^ assay performed better than the RIDA^®^GENE assay for *Giardia* detection, using our extraction technique. The BD Max^TM^ system yielded results in agreement with the 93.5%–100% sensitivity observed in the literature [14, 17, 18]. Interestingly, one sample was negative by microscopy and positive with the three assays. This patient was previously diagnosed with *G. intestinalis*, so this result could either reflect the presence of specific DNA without living parasite, or parasite recrudescence, or resistance to treatment. This highlights that, while molecular techniques are adapted for the diagnosis of intestinal protozoosis, more studies are needed to define their role in post-treatment follow-up.

Regarding *Cryptosporidium* detection, the G-DiaPara^TM^ and RIDA^®^GENE assays showed very good sensitivity and specificity using routine specimens. The BD Max^TM^ assay had a slightly lower sensitivity mainly impacted by PCR inhibitors, leading to 25% (2/8 *C. parvum/hominis*) false-negative results. Despite this, the BD Max^TM^ assay was the only to detect *C. parvum* DNA in a stool negative by microscopy (sample not included in the panel, but tested retrospectively). The patient had been previously diagnosed with cryptosporidiosis and this negative stool was sampled before the onset of antiparasitic treatment, thus this could rather be a “true positive” undetected by microscopy. A possible explanation for this apparently variable performance might be that the BD Max^TM^ system performs better than the MagNA Pure 96 system for nucleic extraction, although it is more susceptible to PCR inhibitors. Interestingly, the BD Max^TM^ and G-DiaPara^TM^ assays detected *C. meleagridis* DNA, whereas they are designed to detect only *C. parvum/hominis*, which could be explained by the fact that these species are genetically close. Such cross-detection is not systematic, as in the study of Laude et al. a stool positive for *C. meleagridis* was undetected by the G-DiaPara^TM^ assay [12]. Finally, even though *C. parvum/C. hominis* are the species mainly responsible for cryptosporidiosis worldwide, the use of PCR assays restricted to these species should be followed by microscopic examination of stools, if the PCR result is negative. In fact, in France, species other than *C. parvum/C. hominis* account for about 10% of cases [4], which would remain undiagnosed by these assays.

Detection of *E. histolytica* was entirely concordant among the three assays. Results were coherent in that the positive sample came from a patient who was positive for *E. histolytica/dispar* by microscopy. This patient had another stool positive by PCR for *E. histolytica*, but negative by microscopic examination, suggesting that microscopy missed one positive sample. Today, molecular biology is critical in the diagnosis of *E. histolytica* and allows us to distinguish real cases of amoebiasis from carriage of *E. dispar*, *E. moshkovskii* or *E. bangladeshi*, which are poorly detected using antigenic assays [3, 20, 23, 24]. This discrimination is crucial to rationalize drug consumption and to avoid misdiagnosis.

The detection of *D. fragilis* by the RIDA^®^GENE assay showed a sensitivity of 71%, and was mainly affected by low parasitic loads. Diagnosis of dientamoebiasis by microscopy is highly difficult, principally because trophozoites are pleomorphic and degrade rapidly outside the intestinal lumen. Therefore, PCR is considered to be the reference method for *D. fragilis* detection [22]. However, available molecular assays are at risk of false positivity because of cross-reactions [6]. In our cohort, two samples were positive whereas microscopic examination of the stool sample was negative and these patients had no previous history of dientamoebiasis. These samples contained *Pentatrichomonas hominis* and *Chilomastix mesnili*, two flagellated protozoa, which are likely to induce cross-reactions. Although the stool containing *P. hominis* was unique in this cohort, other stools contained *C. mesnili*, sometimes in high quantity, and did not yield false-positive results by the RIDA^®^GENE assay. This suggests that *P. hominis* could be responsible for cross-reactions, which is concordant with the fact that, unlike *C. mesnili*, it belongs to the Trichomonada, together with *D. fragilis*. Nevertheless, the only way to confirm this hypothesis would be to sequence PCR products obtained with the RIDA^®^GENE assay primers targeting *D. fragilis*. As the pathogenicity of *D. fragilis* remains debated [22, 25], the need to include this protozoa in multiplex PCR panels may be questionable. However, even though the relationship between *D. fragilis* carriage and diarrhoea is unclear, reports of gastrointestinal symptoms related to heavy parasitic loads are frequent [22]. Furthermore, many studies on *D. fragilis* pathogenicity relied on microscopic examination of stools, which is not an ideal method as discussed above, and could impact the strength of the observed association between parasite carriage and symptoms. For these reasons, molecular biology could add value for further epidemiological studies on the pathogenicity of this protozoon.

The most striking observation in this study is the heterogeneous sensitivity, especially for *G. intestinalis* detection. The employed DNA extraction process could be insufficient in this respect, explaining the lower performances of the G-DiaPara^TM^ and RIDA^®^GENE assays. For example, Adamska et al. compared various methods for extraction of DNA from *G. intestinalis* cysts and concluded that each stage before amplification was crucial [2]. They also reported their best results with an overnight proteinase K treatment instead of short incubations. While some studies reported better DNA extraction of *G. intestinalis* after a bead-beating step of stools, many others observed similar results without mechanical lysis [7, 10, 12, 24]. Interestingly, some authors also reported poorer DNA extraction after this step, depending of the extraction technique used and on the type of beads. For example, in the study of Kaisar et al. [11], mechanical lysis diminished the number of positive samples for *G. intestinalis* and *D. fragilis*. In the same way, a multicentric study showed highly variable effects of the bead-beating step on the extraction of *Cryptosporidium* DNA, especially with the MagNA Lyser^®^ Green Beads which impacted it negatively [16]. The other major factor is the extraction device itself. In the literature, few studies employed the MagNA Pure 96 for protozoa detection. The most interesting one is that of Gotfred-Rasmussen et al., describing sensitivity of 91% for *Giardia* detection by an in-house PCR, compared to direct fluorescent assay. [9] No pre-treatment was applied, except incubation in a transport medium. The extraction reagent was the DNA and Viral NA Large Volume, which enables treatment of higher sample input than the Small Volume reagent we used.

Finally, sensitivity could also be affected by PCR inhibitors. Internal control DNA is supposed to detect them, but using the recommended volumes, the Ct of amplification was always low, thus unlikely to be able to detect moderate inhibition. However, a dilution to 1:10 of DNA extracts from false-negative samples did not allow us to recover the positivity of any sample, which seems to rule out the hypothesis that PCR inhibitors would be the source of false-negative results. Another hypothesis could be that long storage at −80 °C (up to one year before extraction) could have altered DNA and impact performances. However, the BD Max^TM^ assay detected most *Giardia* sp. positive stools, showing that DNA quality was unlikely an issue.

Basically, no assay showed a sufficiently outstanding performance for the detection of all parasites in routine use to discard microscopic examination of stools. Although multiplex PCR techniques would be of great interest in diagnostic labs with automated platforms, more studies are needed to standardize procedures for DNA extraction, which is the critical step. Furthermore, the pathogens targeted by these assays are the most common and most pathogenic protozoa and fit with 90% of screened patients in routine diagnosis, but the sole use of these assays could lead to the non-diagnosis of other protozoa such as *Cystoisospora belli*, *Cyclospora cayetanensis*, and *Blastocystis hominis*. Moreover, no marketed multiplex PCR assay targets helminths, which rules out the possibility of abandoning microscopy. Nevertheless, a reliable molecular assay for protozoa detection would lighten microscopic examination of stools, as helminths are easily detected. In this context, particularly for laboratories with a strong migrant clientele, single tests with enlarged parasitic panels would be welcome.
